# Raman Spectroscopic Analyses of Jaw Periosteal Cell Mineralization

**DOI:** 10.1155/2017/1651376

**Published:** 2017-01-23

**Authors:** Eva Brauchle, Daniel Carvajal Berrio, Melanie Rieger, Katja Schenke-Layland, Siegmar Reinert, Dorothea Alexander

**Affiliations:** ^1^Fraunhofer Institute for Interfacial Engineering and Biotechnology (IGB), Department of Cell and Tissue Engineering, Nobelstr. 12, 70569 Stuttgart, Germany; ^2^Department of Women's Health, Research Institute for Women's Health, Eberhard Karls University, Tübingen, Silcherstr. 7/1, 72076 Tübingen, Germany; ^3^Department of Oral and Maxillofacial Surgery, University Hospital of Tübingen, 72076 Tübingen, Germany; ^4^Department of Medicine/Cardiology, Cardiovascular Research Laboratories, David Geffen School of Medicine at UCLA, 675 Charles E. Young Drive South, MRL 3645, Los Angeles, CA, USA

## Abstract

To achieve safer patient treatments, serum-free cell culture conditions have to be established for cell therapies. In previous studies, we demonstrated that serum-free culture favored the proliferation of MSCA-1^+^ osteoprogenitors derived from the jaw periosteum. In this study, the in vitro formation of bone-specific matrix by MSCA-1^+^ jaw periosteal cells (JPCs, 3 donors) was assessed and compared under serum-free and serum-containing media conditions using the marker-free Raman spectroscopy. Based on a standard fluorescence assay, JPCs from one patient were not able to mineralize under serum-containing culture conditions, whereas the other cells showed similar mineralization levels under both conditions. Raman spectra from mineralizing MSCA-1^+^ JPCs revealed higher levels of hydroxyapatite formation and higher mineral to matrix ratios under serum-free culture conditions. Higher carbonate to phosphate ratios and higher crystallinity in JPCs cultured under serum-containing conditions indicated immature bone formation. Due to reduced collagen production under serum-free conditions, we obtained significant differences in collagen maturity and proline to hydroxyproline ratios compared to serum-free conditions. We conclude that Raman spectroscopy is a useful tool for the assessment and noninvasive monitoring of in vitro mineralization of osteoprogenitor cells. Further studies should extend this knowledge and improve JPC mineralization by optimizing culture conditions.

## 1. Introduction

Jaw periosteum-derived osteoprogenitor cells (JPCs) represent an optimal stem cell source for bone tissue engineering applications in oral and maxillofacial surgeries. Detailed characterization of JPCs, optimized serum-free culture, and differentiation conditions must be established to license these cells for patient studies. Cell populations extracted from the jaw periosteum tissues are heterogeneous. We showed previously that a subpopulation of JPCs expressing a high level of mesenchymal stem cell antigen-1 (MSCA-1) was shown to exhibit an increased osteogenic potential compared to the MSCA-1^low^ cell fraction [1]. In order to avoid immunological reactions in future clinical studies, we established serum-free culture conditions and observed that the proliferation of the MSCA-1^+^ cell fraction was favored in serum-free medium [2]. At the same time, we detected an earlier but weaker mineralization potential of serum-free cultured JPCs, which might significantly counter the success of future tissue engineering applications.

In order to evaluate the calcium phosphate precipitates formed by cells, staining procedures such as Alizarin Red S, von Kossa, and OsteoImage® are commonly used [2–4]. These approaches are able to quantify mineral deposition but fail to assess the quality of the mineralized species. Raman spectroscopy overcomes this limitation by detecting vibrational modes of molecules. Fingerprint Raman spectra have been described to represent a nondestructive readout for the identification of cell phenotypes [5], grading and assessment of malignancy [6–8], and analysis of extracellular matrix components [9, 10]. The detection and capturing of circulating tumor cells were demonstrated to represent further application modalities for the Raman technology [11, 12]. In mineralized tissues, Raman spectroscopy is sensitive to mineral lattice structures of phosphate and carbonate. Furthermore, it can detect vibrations derived from the collagenous matrix. The technique can give comprehensive insight into the biochemical composition and structure of bone and the contribution of various matrix proteins on bone material properties [13]. Raman spectra in a window from 400 cm^−1^ to about 1800 cm^−1^ include the most characteristic phosphate and carbonate bands that represent bone mineral and bone matrix components [13]. The most prominent phosphate band is detected at ~961 cm^−1^, whereby the exact position is sensitive to monohydrogen phosphate (HPO_4_) [13]. Newly formed bone has been shown to exhibit a high HPO_4_ content resulting in a Raman band shifted to lower wavenumbers [13, 14]. The reported positions of the major carbonate and apatite peaks vary as a function of age, health status, and interindividual variations between human samples [13]. Proteins of bone are represented in the Raman spectrum in the phenylalanine band at 1005 cm^−1^, proline (856 cm^−1^), hydroxyproline (881 cm^−1^), and amide III (1242–1280 cm^−1^) as well as the amide I band between 1660 and 1690 cm^−1^. Based on these Raman peaks as well as the phosphate and carbonate specific signals, qualitative readouts of mineralization can be given by calculating mineral to matrix ratios, carbonate to phosphate ratio, and crystallinity [13]. As protein signals are sensitive to biochemical characteristics of collagen, spectra can be used to investigate collagen maturity [13, 15].

In this study, we employed Raman microspectroscopy to assess the osteogenic differentiation and the biochemical composition of mineralized species formed by JPCs under different media conditions. JPCs derived from the jaw periosteum of three male patients were magnetically separated for MSCA-1^+^ expression prior to differentiation. We induced in vitro osteogenesis of these cells under serum-containing and serum-free culture conditions (DMEM versus MesenCult (MC) medium) and collected Raman spectra from both cultures after 5 and 20 days of differentiation. Using a multivariate approach, we compared the mineralization content based on the spectra obtained. Furthermore, qualitative measures of the mineralized matrix formed in vitro were derived from Raman spectra and compared under both media conditions.

## 2. Materials and Methods

### 2.1. Cell Isolation and Culture

After obtaining written informed consent, JPCs derived from 4 donors (3 males: age 21 (donor #1 with complex fractures of the midface, referred to as healthy), 60, 74, and 81 (donor #2, carcinoma of the floor of the mouth; #3, carcinoma of the buccal side; #4, carcinoma of the alveolar process)) were included in this study in accordance with the local ethical committee (approval number: 194/2008BO2). Jaw periosteal tissue from donors #2, 3, and 4 was extracted from an area in safe distance to the identified tumor. Therefore, the jaw periosteal tissues were mechanically broken up, followed by an enzymatic digestion using type XI collagenase (1500 U/ml, Sigma-Aldrich, Steinheim, Germany) for 90 min. Afterwards, the JPCs were plated into 75 cm^2^ culture flasks. For JPC expansion, cells were cultured in DMEM/F-12 (Invitrogen-BioSource Europe, Nivelles, Belgium) containing 10% FCS (Sigma-Aldrich, Steinheim, Germany) and 1% fungicide and penicillin/streptomycin (Biochrom, Berlin, Germany). DMEM-cultured cells were passaged using Trypsin-Versene EDTA (1x, Lonza, Basel, Switzerland). After stepwise reduction of the FCS content in the cultures, medium was then switched to the serum- and xeno-free medium MesenCult-XF (MC-XF, STEMCELL Technologies, Vancouver, Canada) and cells were passaged using the MesenCult-ACF dissociation kit. Furthermore, for MC-XF culture conditions, culture dishes or flasks were coated overnight with MesenCult-XF attachment substrate provided by the same company.

For Raman analyses, serum-containing and serum-free MSCA-1^+^ JPC cultures (derived from donors #1, #2, and #3) were all assessed in coated (MesenCult-XF attachment substrate) glass bottom dishes (CELLview™ cell culture dishes from Greiner Bio-One GmbH, Germany). For Raman measurements, the used JPCs were not fixed but measurements were carried out on living cells covered by cell culture medium. MSCA-1^+^ JPC cultures derived from #4 were used beside the other three donors for gene expression analyses (quantitative PCR).

### 2.2. Magnetic Cell Separation of the MSCA-1^+^ Cell Fraction by MACS

The entire JPC population underwent magnetic separation in order to isolate the MSCA-1^+^ cell fraction as described before [16]. In brief, JPCs were incubated with the FcR blocking reagent (400 *µ*l) and the anti-MSCA-1 MicroBeads (400 *µ*l, Miltenyi Biotec, Bergisch Gladbach, Germany) for 20 min at 4°C. This monoclonal antibody reacts with the tissue nonspecific alkaline phosphatase [17]. After washing, the samples were applied to preseparation filters, thereafter onto MS columns. The MSCA-1^+^ cells remained within the column in the magnetic field. After removing the column from the magnetic field, MSCA-1^+^ fraction could be eluted.

For the switchover from serum-containing to serum-free media conditions, MSCA-1^+/-^ cell fractions underwent a stepwise serum reduction and cells were then cultured in MesenCult-XF medium (MC-XF, STEMCELL Technologies, Grenoble, France) containing 1% glutamine and 1% fungicide and penicillin/streptomycin, as already described elsewhere [2]. For Raman spectroscopic analyses, MSCA-1^+^ cell fractions were cultured under FCS-containing (DMEM) and animal-free culture conditions (MC).

### 2.3. Osteogenic Differentiation

**Figure 1:**
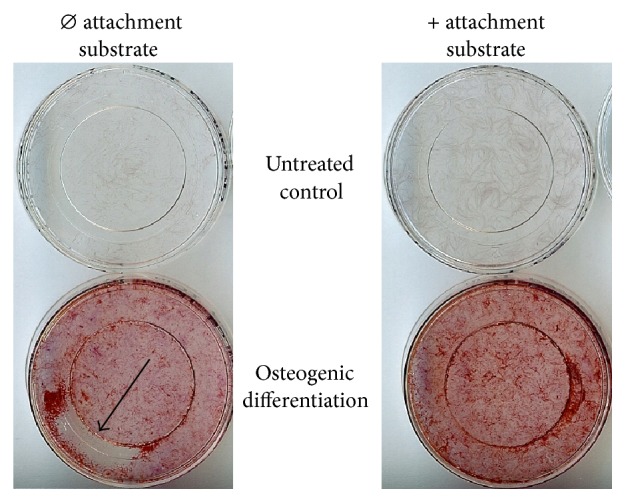
JPCs were seeded into uncoated/coated glass bottom dishes under untreated and osteogenic conditions. MSCA-1^+^ JPCs were seeded into uncoated/coated glass bottom dishes under untreated and osteogenic conditions. Plates without attachment were unproblematic for untreated JPCs. On the basis of increased detachment of osteogenically induced monolayers, glass bottom dishes coated with MesenCult-XF attachment substrate were used for all Raman measurements.

The DMEM-cultured MSCA-1^+^ cell fractions were seeded at a density of 4 × 10^4^ cells and the MC-cultured cell fraction was seeded at a density of 6 × 10^4^ cells per CELLview culture dish with integrated glass bottom (Greiner Bio-One, Frickenhausen, Germany). For all Raman analyses, CELLview culture dishes were precoated with MesenCult-XF attachment substrate for better cell adherence. Preliminary tests revealed low cell adherence with partial cell detachment without precoating (Figure 1) particularly for osteogenically induced cells. For osteogenic differentiation, MSCA-1^+^ cell fractions were treated with osteogenic medium (ob, DMEM/F12 containing 10% FCS, 10 mM *β*-glycerophosphate, 100 *µ*M L-ascorbic acid 2-phosphate, and 4 *µ*m dexamethasone, Sigma-Aldrich) for 20 days.

Xeno-free (MC-) cultured MSCA-1^+/*‒*^ cell fractions were osteogenically induced using the MesenCult osteogenic medium (ob), containing MesenCult MSC basal medium, 5% osteogenic stimulatory supplement, and 3.5 mM ß-glycerophosphate, as described before [2] for the same time period as DMEM-cultured cells. For both, serum-free and serum-containing culture conditions, untreated and undifferentiated controls (co) were simultaneously cultured.

### 2.4. Raman Microspectroscopy and Data Analysis

A custom-made Raman microspectroscope, as previously described, was employed for all measurements [18]. Briefly, the system is composed on a commercial fluorescence microscope (IX71 Olympus, Japan). Raman spectra were excited by guiding a 785 nm laser beam (TOPTICA AG, Munich, Germany) through a 100x oil-immersion objective (NA 1.4, Olympus). The system was calibrated based on the silicon peak at 522 cm^−1^ prior to all measurements. The laser output power was set on 85 mW for spectra acquisition. Raman spectra were collected from either MSCA-1^+^ JPCs cultured with serum-containing media (DMEM) or MSCA-1^+^ JPCs cultured under serum-free conditions (MC) after 5 and 20 days of osteogenic differentiation. All cells were cultured in coated glass bottom cell culture dishes (Greiner Bio-One GmbH, Germany). The total acquisition time per spectrum was 100 seconds. A background spectrum from the glass dish containing the cell culture medium was taken for each set of data. All acquired Raman spectra were background-subtracted using the specific background spectrum and then baseline-corrected (rubber-band-method, 64 data points) using OPUS (Bruker Optics, Billerica, MA, USA). A smoothing algorithm (Savitzky-Golay, second polynomial order, 7 data points) was employed on all spectra. Then, multivariate PCA models were calculated to compare Raman data after 5 and 20 days of differentiation for each patient using Unscrambler ×10.3 (CAMO Software, Oslo, Norway) as previously described [10]. All principal components (PCs) were screened for apatite signals at 961 cm^−1^. In each PCA, PC loadings showing apatite signals were identified and selected. Score values for selected PCs were plotted and depicted as three-dimensional scatter plots using OriginPro 2015.

Raman spectra were further employed to compare the mineral composition that was formed under DMEM- and MC-cultured conditions. The following spectral ratios were calculated from the Raman spectra: hydroxyapatite (HA) to phenylalanine (961/1005 cm^−1^), HA to amide III (961/1244 cm^−1^), and carbonate to HA (1070/961 cm^−1^). As a measure of the HA crystallinity, the inverse of the full width at half maximum (FWHM) was calculated for the spectral range from 900 to 1000 cm^−1^ using MATLAB (MathWorks, Natick, USA). The range was isolated and detrending was performed. The apatite peak was then fitted using a standard Gauss curve. Based on the fitted curve, the inverse of FWHM (1/FWHM) was calculated for the HA peak at 961 cm^−1^.

### 2.5. Gene Expression Analysis in DMEM- and MC-XF-Cultured JPCs by Quantitative PCR

RNA isolation from MSCA-1^+^ JPCs (*n* = 4 for each group) cultured under serum-containing and serum-free conditions was carried out using the NucleoSpin RNA XS kit (Macherey-Nagel, Düren, Germany) following the manufacturer's instructions. After photometrical determination of RNA concentrations (GeneQuant Pro; GE Healthcare), 15 ng of RNA was synthesized to cDNA using the QuantiTect Whole Transcriptome Kit (Qiagen, Venlo, Netherlands) following the manufacturer's instructions.

Messenger-RNA transcription levels were quantified using the real-time LightCycler System (Roche Diagnostics, Mannheim, Germany). For the PCR reactions, commercial primer kits (Search LC, Heidelberg, Germany) and DNA Master SYBR Green 1 (Roche) were used. 35 cycles were used for amplification of the target mRNAs. The ratios of type I collagen (*α*1 and *α*2 chains) transcript numbers to the corresponding housekeeping glyceraldehyde-3-phosphate dehydrogenase (GAPDH) gene levels were calculated and illustrated in Figure 7.

### 2.6. Statistical Analysis

Raman spectra from each donor were averaged. Spectra from each donor were used to calculate means for Raman spectral ratios. A two-tailed ANOVA was employed (Prism, GraphPad Software, La Jolla, USA) to identify statistical differences of bone formed under DMEM and serum-free MC conditions for all donors (*n* = 3, *p* < 0.001, for all tests).

For the evaluation of the PCR data, means ± standard deviations are expressed and, for the statistical analysis, two-tailed *t*-tests were used. A *p* value < 0.05 was considered significant.

## 3. Results

### 3.1. Identification of Suitable Glass Bottom Plates for the Performance of Raman Measurements

JPCs were seeded into precoated or uncoated glass bottom dishes under untreated or osteogenic conditions. As illustrated in Figure 1, particularly osteogenically induced cell monolayers detached occasionally in uncoated glass dishes. Therefore, we decided to coat the dishes for all Raman measurements and for both analyzed culture conditions (serum-containing and serum-free conditions).

### 3.2. Raman Spectra Indicate Osteogenic Differentiation in MSCA-1^+^ JPCs

**Figure 2:**
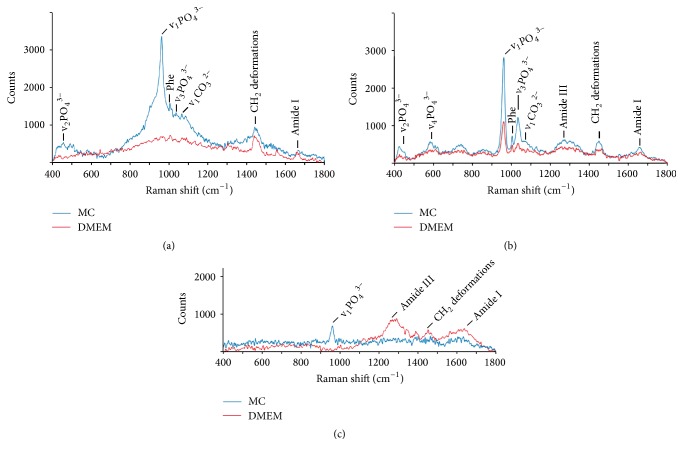
Mean Raman spectra collected from MSCA-1^+^ JPCs after 20 days of osteogenic differentiation in DMEM and MC medium. (a) Spectra from patient #1: averages were generated from 79 (MC) and 48 (DMEM) single spectra; (b) 128 (MC) and 37 (DMEM) single spectra were averaged for mean spectra from patient #2; (c) mean spectra from patient #3 were generated from 46 (MC) and 100 (DMEM) single spectra. Bone-specific peaks were assigned based on Mandair and Morris (2015) [13].

Raman spectra from MSCA-1^+^ JPCs after 5 days of differentiation showed a high patient variability and low signal to noise ratios, which made it difficult to assign peaks (Supplemental Figure 1 in Supplementary Material available online at https://doi.org/10.1155/2017/1651376). After 20 days of differentiation, Raman spectra of JPCs from patient #1 showed typical modes of hydroxyapatite vibrations at 430 (v_2_PO_4_^3−^), 961 (v_1_PO_4_^3−^), and 1045 cm^−1^ (v_3_PO_4_^3−^) in MC medium. In contrast, when these cells were differentiated in DMEM media, clear contributions of hydroxyapatite were not observed based on the mean spectra (Figure 2(a)). In Raman spectra from patient #2, all vibrational modes of hydroxyapatite were detected, including 590 cm^−1^ (v_4_PO_4_^3−^), indicating a higher degree of mineral deposition compared to MC-cultured JPCs from patient #1. In DMEM-cultured JPCs from patient #2, apatite formation was also detected. The intensity of the hydroxyapatite signals in DMEM-cultured JPCs from patient #2 was lower compared to spectra from the same patient cultured under MC conditions (Figure 2(b)). Due to overlay with v_3_PO_4_^3^ peak, the carbonate vibration v_1_CO_3_^2−^ at 1070 cm^−1^ was detected as a peak shoulder in spectra from patient #1 and patient #2 under both media conditions. Raman spectra from patient #3 showed a lower signal intensity and thus lower signal to noise ratio for both conditions compared to the spectra from JPCs of patient #1 and patient #2. In MC-cultured JPCs from this patient, v_1_PO_4_^3−^ at 961 cm^−1^ was detected, whereas more prominent amide vibrations and CH deformations from proteins were detected in the spectra under DMEM conditions (Figure 2(c)).

### 3.3. Fluorescence Detection of Mineralization and Multivariate Analysis of Raman Spectra

**Figure 3:**
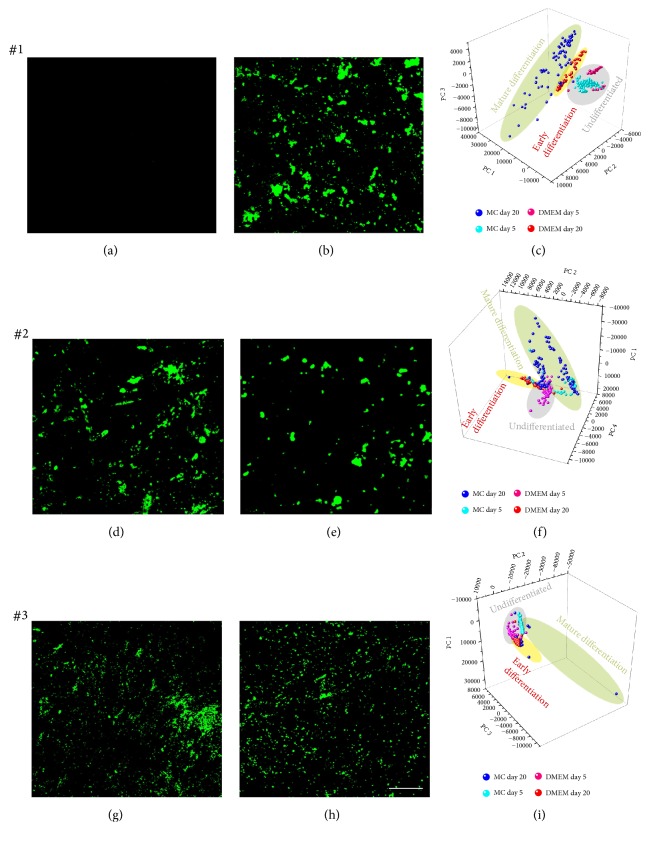
Fluorescence staining of hydroxyapatite and PCA of Raman spectra from differentiated MSCA-1^+^ JPCs. (a, b, d, e, g, h) OsteoImage fluorescence staining and (c, f, i) PCA scores plots of JPCs isolated from (a–c) patient #1, (d–f) patient #2, and (g–i) patient #3 after differentiation in (a, d, g) serum-containing DMEM and under (b, e, h) serum-free conditions (MC) for 20 days. Scale bar equals 200 *µ*m. (c, f, i) PCA plots of Raman spectra using PCs shown in Figure 4. The PCAs reflect a more mature bone formation (green ellipse) under MC media conditions compared to DMEM conditions (yellow ellipse) after 20 days of differentiation for all patients.

**Figure 4:**
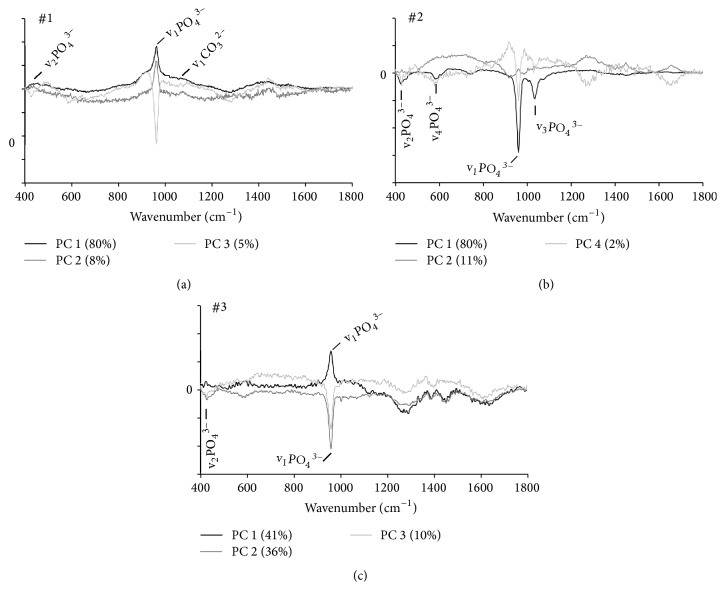
Loadings of PCA describe the mineralization for each patient. (a) PC 1, PC 2, and PC 3 loadings demonstrate strong influences of hydroxyapatite peaks (v_2_PO_4_^3−^, v_1_PO_4_^3−^) at 961 and 430 cm^−1^ in the PCA of Raman spectra from patient #1 (Figure 3(a)). (b) PC 1, PC 2, and PC 4 loadings referring to the PCA of patient #2 (Figure 3(b)) exhibit the four major hydroxyapatite vibrations. (c) Loadings of PC 1, PC 2, and PC 3 validate that the Raman spectra of patient #3 exhibit signals from hydroxyapatite.

Osteogenic differentiation was assessed from MSCA-1^+^ JPCs cell fractions after 20 days in either DMEM or MC media by fluorescence staining of hydroxyapatite (OsteoImage). Hydroxyapatite was not detected in MSCA-1^+^ JPCs from patient #1 cultured in DMEM media (Figures 3(a) and 3(b)), whereas cells from patient #2 and #3 formed hydroxyapatite deposits under DMEM culture conditions (Figures 3(d) and 3(g)). In MC media, MSCA-1^+^ JPCs from all patients formed hydroxyapatite crystals (Figures 3(b), 3(e), and 3(h)). To analyze the heterogeneous Raman spectral data sets and to visualize differences in mineralized species for DMEM- and MC-cultured JPCs, PCA was employed on the spectra. PCA extracts PC score values and loading spectra, which describe the spectral variances in the data set and demonstrate the variability of single Raman spectra for each cell culture condition. The method can identify differences in spectra due to different stages of matrix maturation in DMEM- and MC-cultured MSCA-1^+^ JPCs. Due to high donor variability, Raman data from DMEM- and MC-cultured MSCA-1^+^ JPCs of each donor were analyzed in a separate PCA model. By plotting PCA scores of PC 1, PC 2, and PC 3, it was confirmed that Raman spectra from differentiated MC- and DMEM-cultured MSCA-1^+^ JPCs from patient #1 could be separated from undifferentiated cultures. Whereas a more mature bone matrix was formed under MC-conditions, PCA scores also indicate an early (immature) stage of mineralization in DMEM-cultured JPCs after 20 days of differentiation (Figure 3(c)). Loadings of PC 1, PC 2, and PC 3 exhibited a strong hydroxyapatite peak (Figure 4(a)), which confirms that PCA score values reflect different degrees of mineralization. In MSCA-1^+^ JPCs of patient #2, PCA scores identified differences between MC- and DMEM-cultured cells after 20 days of differentiation (Figure 3(f)). In this PCA, Raman signals from hydroxyapatite were reflected predominantly in PC 1 and PC 4 loadings (Figure 4(b)). PCA of cells from patient #2 indicates again an earlier (immature) stage of differentiation under DMEM conditions compared to MC conditions (Figure 3(f)). Under both media conditions, Raman spectra from differentiated cells from patient #3 showed decreased intensities at hydroxyapatite positions compared to JPCs from other patients. This finding might correlate to the fact that differentiated MSCA1^+^ JPCs from patient #3 formed smaller hydroxyapatite crystals (Figure 3(g)). PC 1, PC 2, and PC 3 loading depicted a strong impact of the hydroxyapatite peak for the data from patient #3 (Figure 4(c)). JPCs differentiated under DMEM and MC conditions for 20 days showed a population, which is slightly separated from undifferentiated cells; thus PCA indicates a contribution of mineralized species to spectra from JPCs differentiated under both DMEM and MC media. PCA demonstrated that few spectra of MC-cultured JPCs of patient #3 exhibit strong mineralization (Figure 3(f)).

### 3.4. Assessment of Bone Quality Formed by In Vitro Differentiated MSCA-1^+^ JPCs

**Figure 5:**
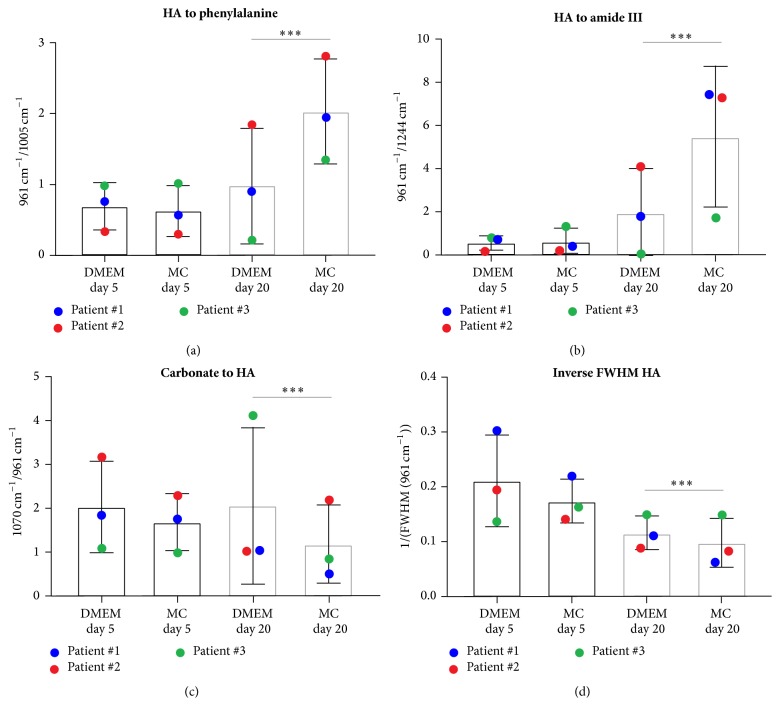
Spectral ratios to assess the mineral compositions of DMEM- and MC-cultured MSCA-1^+^ JPCs in osteogenic differentiation. Mineral to matrix ratio as indicated by (a) ratio of hydroxyapatite (HA) to phenylalanine signal and (b) ratio of HA to amide III. Both ratios are significantly increased under MC conditions after 20 days of differentiation. (c) Carbonate to HA (1070/961 cm^−1^) ratios were significantly higher in JPCs differentiated under DMEM conditions. (d) The crystallinity is indicated by the inverse of full width at half maximum (FWHM) of the HA peak and was significantly decreased under serum-free MC culture conditions compared to DMEM conditions indicating more mature differentiation. Asterisks indicate statistical significances: ^*∗∗∗*^*p* < 0.001.

Raman peak ratios were employed to compare the quality of the mineralized matrix formed by MSCA1^+^ JPCs under DMEM and MC conditions. By analyzing the ratio between the Raman spectral intensities of hydroxyapatite and phenylalanine (Figure 5(a)) and hydroxyapatite to amide III peaks (Figure 5(b)), it was evident that bone matrix formed by JPCs exhibits significantly lower mineral to matrix ratios under DMEM conditions compared to JPCs differentiated under MC conditions. Moreover, an increase in mineral to matrix ratio was detected for MC-cultured JPCs when comparing cultures from day 5 and day 20 (*p* < 0.0001). DMEM-cultured JPCs did not change significantly in mineral to matrix ratio over culture time (Figures 5(a) and 5(b)). In contrast, carbonate to phosphate ratios were significantly higher in cells cultured under DMEM media conditions (Figure 5(c)). The inverse of the FWHM of the 961 cm^−1^ hydroxyapatite peak is related to crystal size or crystallinity [13, 19]. DMEM-cultured JPCs exhibited significantly higher crystallinity and thus cells formed larger crystals compared to MC cultures, indicating a more distinct hydroxyapatite peak in DMEM-cultured JPCs (Figure 5(d)) with few contributions of immature mineral components such as hydrogen phosphate [20].

**Figure 6:**
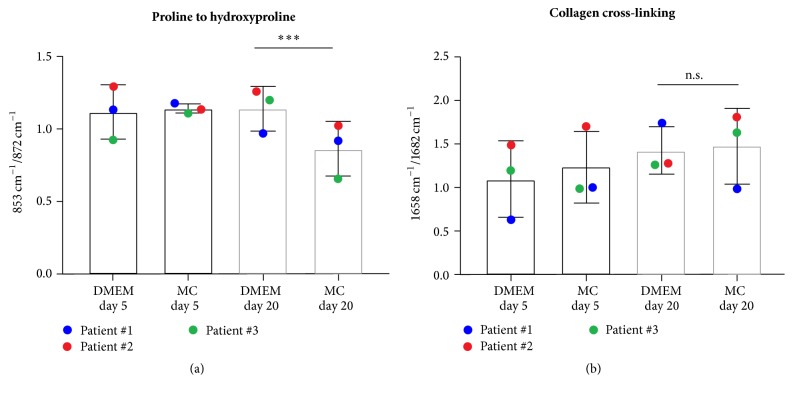
Spectral ratios to assess the maturity degree of collagen formed at mineralized sites in DMEM and serum-free cultured MSCA-1^+^ JPCs. (a) Proline to hydroxyproline ratio (853/872 cm^−1^) indicating significant differences in maturation of collagen fibrils due to media conditions after 20 days of differentiation (statistical significance is indicated by asterisk: ^*∗∗∗*^*p* < 0.001). (b) Collagen cross-linking calculated from the 1658/1682 cm^−1^ ratio (n.s., not statistically significant).

The collagen network, which is necessary for the initiation of apatite crystal formation, was further assessed by investigating collagen-specific measures in Raman spectra of the overall data sets. The amide I peak, which has been described to be reflective for differences between newly formed and mature collagen fibers [19, 21], indicated that collagens formed by MSCA1^+^ JPCs under MC and DMEM conditions differ in their degree of maturity (Figures 6(a) and 6(b)). Proline to hydroxyproline ratios were significantly lower in MC-cultured JPCs, strengthening the hypothesis that serum-free MC media support mineralization via maturation of collagens in JPCs. Supplemental Figure 2 shows representative mean Raman spectra of DMEM- and MC-cultured JPCs from patient #2, reflecting the detected differences concerning the collagen maturity.

### 3.5. Type I Collagen Gene Expression Analyses

Based on the fact that type I collagen represents the main collagenous component of the bone, we analyzed gene expression levels in serum-free and serum-containing conditions cultured JPCs.

After normalization to the housekeeping mRNA levels of GAPDH, significantly higher levels of the *α*1 chain of type I collagen were detected in serum cultured MSCA-1^+^ JPCs in comparison to cells cultured under serum-free conditions (untreated: 110.4 ± 44.8 versus 29.7 ± 26.4; osteogenically induced (10 d): 219.2 ± 81.7 versus 96.5 ± 25.8; *p* < 0.05, *n* = 4).

Significantly higher mRNA levels of the *α*2 chain of type I collagen were also detected in untreated DMEM-cultured JPCs as compared to serum-free cultivated MSCA-1^+^ JPCs (49.8 × 10^3^  ± 17 × 10^3^ versus 22.1 × 10^3^  ± 14.3 × 10^3^; *p* < 0.05, *n* = 4). Differences in gene expression levels of Coll 1 (*α*2) did not reach significant values in osteogenically induced JPCs but a similar tendency was detected (62.7 × 10^3^  ± 24.5 × 10^3^ versus 34.2 × 10^3^  ± 4.3 × 10^3^).

## 4. Discussion

JPCs represent an ideal cell source for tissue engineering approaches for the treatment and regeneration of oral and maxillofacial bone defects. To certify these cells for clinical applications, establishment of animal-free culture conditions has to be addressed. Previously, we described that JPCs show higher proliferation rates and a higher expression of the osteoprogenitor marker MSCA-1 after expansion under serum-free culture conditions [2]. To gain deeper insights into the biochemical characteristics of osteogenesis under serum-free conditions, we compared the mineral composition within the extracellular matrix produced by MSCA-1^+^ JPCs independent of media condition by Raman spectral analysis.

To analyze bone quality and bone formation, ex vivo Raman microspectroscopy has been described as being a powerful technique [13]. Using Raman microspectroscopy, it was shown that the biochemical composition of bone is altered in pathological states such as postmenopausal osteoporosis and osteogenesis imperfecta [19, 21–24]. Furthermore, a correlation of biomechanical properties to Raman spectral information was indicated [25, 26]. Previously, Evans and colleagues used Raman microspectroscopy to compare the biochemical compositions of bone nodules formed by embryonic stem cell, mesenchymal stem cells, and osteoblasts [27]. To the best of our knowledge, this is the first study comparing cell mineralization under standard and serum-free media conditions by employing Raman microspectroscopy. In our study, signals of apatite and carbonate signals in Raman spectra between cells from different donors varied strongly. These interindividual biochemical characteristics of the mineralized species might reflect the degree of matrix maturation. Hung et al. identified that the Raman signal of hydroxyapatite increases with progressive differentiation of mesenchymal cells towards bone and could identify hydroxyapatite, octacalcium phosphate, and ß-tricalcium phosphate Raman signals from the mineralized species at different stages [14]. In our data, differences in hydroxyapatite signal intensity after 20 days of differentiation might reflect donor-specific dynamics of mineral deposition. The intensities of phosphate and collagen peaks are highly dependent on polarization effects [28, 29]. It is therefore possible that different media conditions result in different orientations of mineral crystals and collagen fibers. Polarization effects were not investigated here, since the orientation of apatite crystals formed in vitro was expected to be comparable under both conditions.

PCA is widely used for the analysis of Raman spectra. The use of PCA simplifies the interpretation of highly complex biological Raman spectra by resolving individual biochemical components [30]. Kunstar et al. showed that PC score values can be used to separate spectra from endochondral bone from spectra of the adjacent femur cartilage tissues [31]. In their approach, the most prominent phosphate band at ~961 cm^−1^ was identified in the PC loading spectrum as an indicator of bone tissue. In a similar way, our PC loadings displayed obvious phosphate signals. PCA score values of these PCs were employed to separate spectra from JPCs which were differentiated under DMEM conditions from those differentiated under serum-free conditions. With the exception of MC-cultured JPCs from patient #3, the spectra formed well-defined populations within the scores plot, suggesting a relative low variability of spectral signals within one cell culture dish. The scores plots depict a gradual change of the spectra from day 5 to day 20 for both media conditions and suggest a more immature degree of mineralization for JPCs cultured under serum-containing conditions.

**Figure 7:**
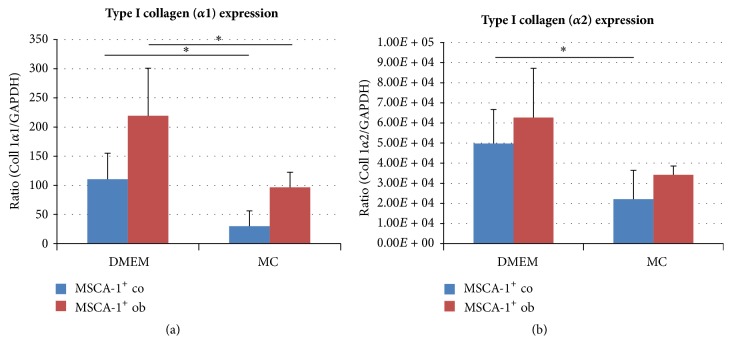
Quantitative PCR measurements of type I collagen (*α*1 and *α*2 chain) gene expression levels in MSCA-1^+^ JPCs cultured under DMEM (serum-containing) and MC conditions (serum-free). (a) mRNA levels of type I collagen (*α*1 chain) and (b) (*α*2 chain) in relation to the housekeeping gene GAPDH are illustrated in untreated (co) and osteogenically induced (ob, for 10 days) MSCA-1^+^ cells. Statistical significances are indicated by asterisks: ^*∗*^*p* < 0.05.

As described by many others [19, 32], we assessed mineral to matrix ratios, carbonate to phosphate ratios, and mineral crystallinity based on our Raman data to compare mineralized cultures after 20 days under serum-containing and serum-free culture conditions. Consistent with our results from PCA analysis of the spectra, mineral to matrix ratios of serum-cultured MSCA-1^+^ JPCs showed similar values after 5 and 20 days of differentiation, indicating that nonmineralized (collagens and proteins) rather than mineralized matrix components (phosphates) were deposited from serum-treated JPCs. However, our previous studies as well as other groups showed that cells mineralize to a higher extent under serum-containing conditions than under serum-free culture conditions [2, 33]. In contrast, in the current study, Raman spectra, PCA, and mineral to matrix ratios demonstrated a more mature bone formation in serum-free cell cultures. These results can be explained by our PCR data (Figure 7) and by previously published data, where unseparated serum-free cultured JPCs expressed significantly decreased levels of type I collagen compared to standard cultures of JPCs [2]. Although the carbonate peak is partially overlapped by the v_3_PO_4_^3−^ at 1045 cm^−1^ in our Raman data, carbonate to phosphate ratios were found to be significantly higher under serum-containing culture conditions, indicating that more carbonate was placed in the hydroxyapatite lattice. A recent work examined bone tissue composition and viscoelastic properties in aging human trabecular bone [34], demonstrating that mineralization decreases with age and carbonate substitution increases [34]. Considering this knowledge, a higher carbonate to phosphate ratio and lower mineral to matrix ratio, as detected under serum-containing differentiation conditions, could be indicative for poor bone quality. In contrast, McManus and coauthors employed Raman spectroscopy to compare the osteogenic differentiation of osteosarcoma cells to primary osteoblasts [35]. They detected significantly higher carbonate to phosphate ratios and lower mineral to matrix ratios in osteosarcoma cells, which might form defective bone structures compared to primary osteoblasts. Roschger and coauthors reported a linear correlation between mineral to matrix ratio measured by Raman spectroscopy and the calcium content in healthy human osteonal bone [36]. However, we deal with an artificial in vitro cell culture model which is most likely not accurately comparable with the osteonal bone.

Mineral crystallinity corresponds to crystal size and was proposed to correlate with the carbonate to phosphate ratio [19, 32]. In our study, mineralization under serum-free conditions exhibited lower carbonate to phosphate levels and smaller hydroxyapatite crystals. Larger mineral crystals indicating higher mineralization capacity were found under serum-containing osteogenic differentiation. Bovine serum contains a lot of active components that have been described to accelerate the crystallization of free phosphates and calcium [37]. Under this aspect, it is not surprising that the carbonate to phosphate ratio is lower under serum-free culture conditions. For our study, we can conclude that serum-free cultured MSCA-1^+^ JPCs form heterogeneous apatite crystals result in a wider hydroxyapatite peak that is generated from immature crystal structures [32]. A similar phenomenon was described for osteogenesis imperfecta [19], showing smaller but more abundant mineral crystals and suggesting that these structural changes might lead to a higher tissue mineral density with lower Young's modulus [19]. In future studies, we are aiming to investigate the impact of serum in media on the elastic modulus and hardness of in vitro formed bone and correlate these data to Raman measures. Biomechanical characteristics of bone tissues are predominantly triggered by the orientation and the structure of collagen fibers [38]. As demonstrated in this study, collagen expression differed in serum-free cultured JPCs from standard cultures. A possible explanation might be the fact that the significantly lower collagen expression in serum-free cultured JPCs correlates to high mineral to matrix ratios and low crystallinity, as seen in osteogenesis imperfecta previously [19]. These structural changes in collagen fibers might impact crystallization of phosphate along the collagen fibrils and could be a trigger to control and guide mineralization.

Previously, it has been shown that substrate stiffness could impact cell differentiation [39]. In our study, JPC adherence on glass bottom dishes was not optimal and, due to this observation, precoating of the plates was performed (Figure 1). However, MSCA-1^+^ JPCs from donor #1 did not deposit hydroxyapatite under serum-containing conditions on coated glass plates, whereas the same cells are able to mineralize within 6-well plates without precoating. Cells from different patients mineralized to a different extent. For both media conditions, Raman spectra from cells of patient #3 exhibited much lower signal to noise ratios. Patient-specific matrix characteristics might influence spectral qualities. This hypothesis needs to be confirmed in future studies, where several culture dishes from one patient should be investigated. Our study highlights the ability of Raman spectroscopy to analyze matrix formation and to compare mineral composition for different culture conditions. Single point Raman measurements as performed in this study are not representative for the mineralization content throughout the whole cell culture dish, which occurs rather locally. Automated scanning of greater defined areas within the cell monolayers can provide Raman images, which might be more representative for the quantification of mineralization content [40].

Optimal substrates for Raman measurements consist of quartz glass or calcium fluoride and generate less background signal in Raman measurements compared to standard glass. On the other hand, due to its hygroscopic characteristics, calcium fluoride is not really stable in water and therefore is probably not suitable for differentiation experiments which take at least 20 days of cell cultivation/differentiation. Raman compatible CaF_2_ slides are more suitable for paraffin-embedded sections or short cell cultivation and/or fixation. In future studies, we will test the suitability of quartz based and attachment substrates dishes for 2D JPC differentiation and imaging. We could circumvent these problems in the future by monitoring of 3D TE constructs, where cells have to grow within the 3D constructs and not on glass bottom surfaces.

In summary, we detected significant differences in the biochemical composition of mineralized species formed by MSCA-1^+^ JPCs under serum-containing and serum-free culture conditions. The more mature bone matrix formed under serum-free culturing probably possesses less elastic properties due to reduced collagen expression. Spectra contain powerful data, which can support the establishment of osteogenic differentiation protocols and the adaption of cultures to serum-free media conditions. The biochemical characterization of mineralized tissues can provide structural and functional information, which might help to classify and discriminate formation of poor quality bone tissue. Moreover, Raman microspectroscopy is suitable to compare in vitro developed engineered tissues with genuine natural tissues in a global molecular-sensitive manner.

## Supplementary Material

Supplemental Figure 1: Raman spectra from MSCA-1+ JPCs after 5 days of differentiation showed a high patient variability and low signal to noise ratios, which made it difficult to assign peaks.
Supplemental Figure 2: shows representative mean Raman spectra of DMEM- and MC-cultured JPCs from patient #2, reflecting the detected differences concerning the collagen maturity.
