# Supplementary material for: Raman Spectroscopic Analyses of Jaw Periosteal Cell Mineralization
Source: Stem Cells Int. 2017 Jan 23;2017:1651376. doi: 10.1155/2017/1651376 (PMC5292402; doi:10.1155/2017/1651376)
Supplement: Supplementary file 1 — Supplemental Figure 1: Raman spectra from MSCA-1+ JPCs after 5 days of differentiation showed a high patient variability and low signal to noise ratios, which made it difficult to assign peaks. Supplemental Figure 2: shows representative mean Raman spectra of DMEM- and MC-cultured JPCs from patient #2, reflecting the detected differences concerning the collagen maturity. [file 1651376.f1.docx]

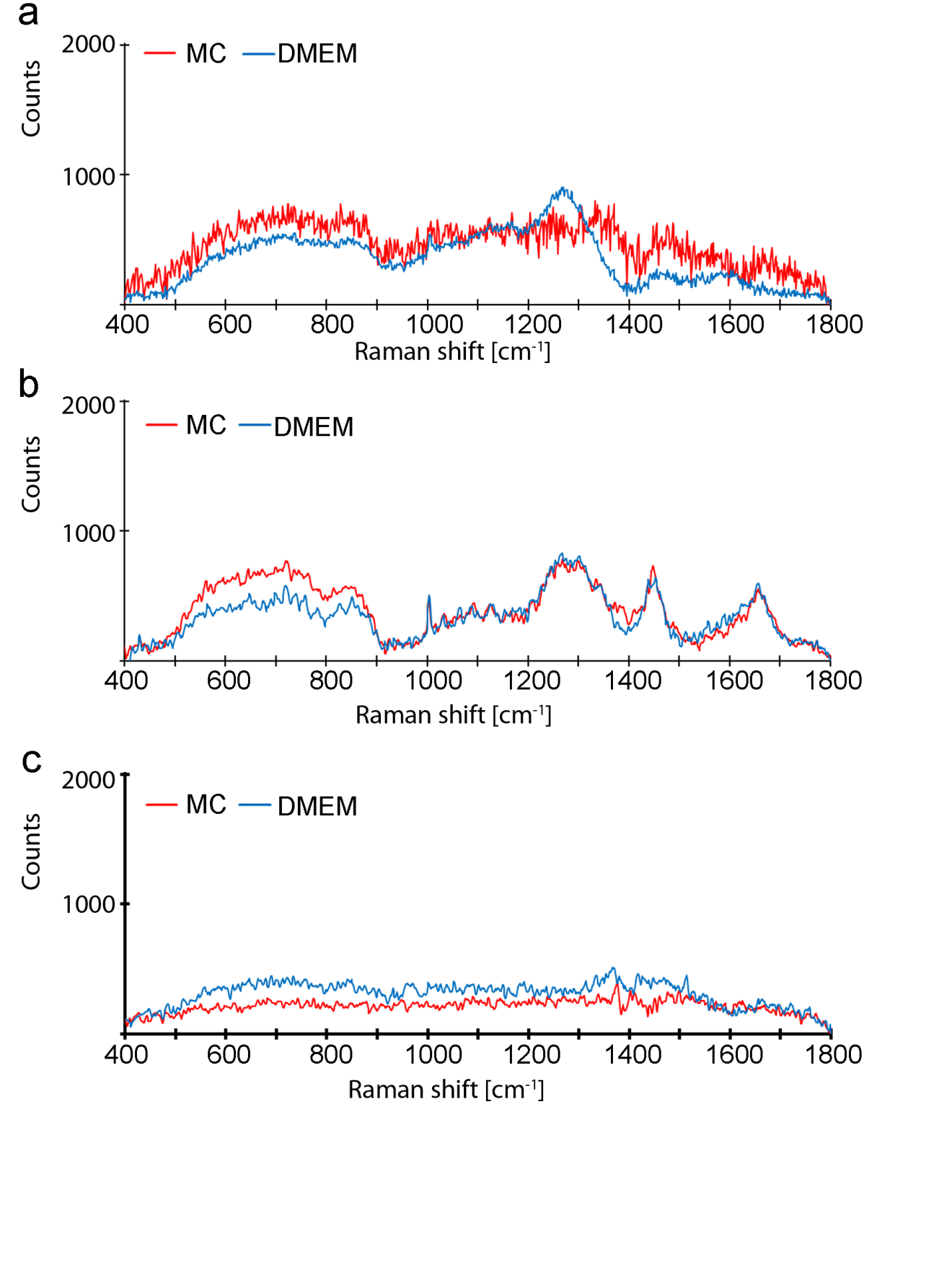


**Supplemental Figure 1.** Mean Raman spectra collected from JPCs after 5 days of
osteogenic differentiation in DMEM- and MC medium. (a) Spectra from patient #1,
averages were generated from 102 (MC) and 47 (DMEM) single spectra, (b) 66 (MC)
and 57 (DMEM) single spectra were averaged for mean spectra from patient #2, (c)
mean spectra from patient #3 were generated from 50 (MC) and 100 (DMEM) single
spectra.


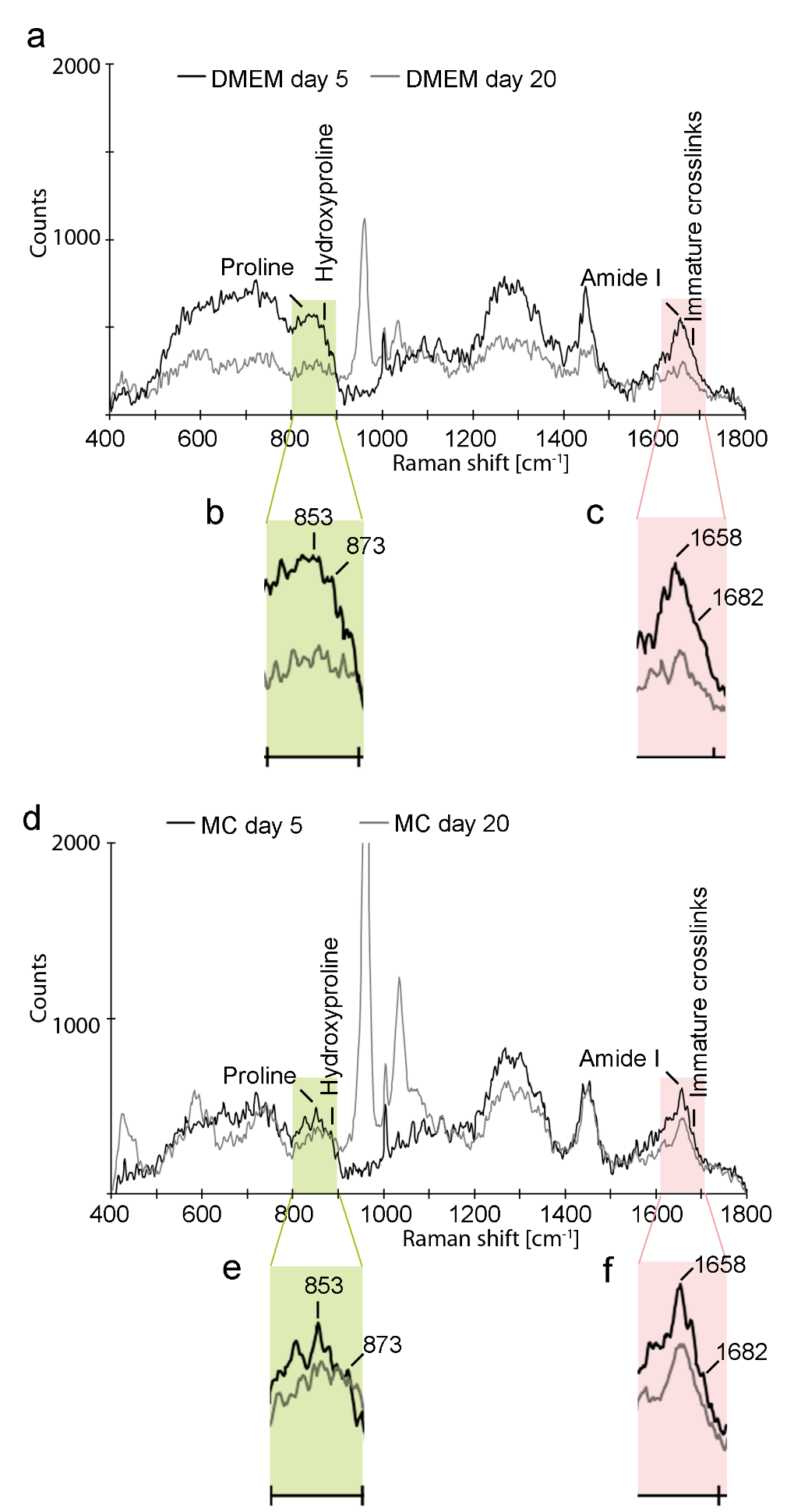


**Supplemental Figure 2:** Mean Raman spectra of a) DMEM-cultured and d) MC-cultured JPCs of patient #2. Spectral areas that are specific for collagen peaks are magnified. (b, e) Proline peak was identified at 853 cm^-1^ and hydroxyproline at 872 cm^-1^. (c, f) Immature collagen crosslinking was detected at 1682 cm^-1^as a shoulder of the Amide I peak at 1658 cm^-1^.
